# Enhancing oncology nursing care planning for patients with cancer through Harnessing large language models

**DOI:** 10.1016/j.apjon.2023.100277

**Published:** 2023-07-26

**Authors:** Abdulqadir J. Nashwan, Salam Bani Hani

**Affiliations:** a Education & Practice Development, Hamad Medical Corporation, Doha, Qatar; b Department of Public Health, College of Health Sciences, QU Health, Qatar University, Doha, Qatar; c School of Nursing, Adult Health Department, Al Al-Bayt University, Mafraq, Jordan

Dear Editor,

Nursing care planning is a critical and daily task in managing and treating patients with cancer. It involves the systematic nursing assessment, diagnosis, planning, interventions, and evaluation of care plans tailored to the unique needs of patients with cancer.^1^^,^^2^ The advent of large language models (LLMs) signifies a leap in the field of natural language processing. These advanced models, like the generative pre-trained transformer-4 (GPT-4) developed by OpenAI, can generate human-like text based on the input they receive. GPT-4 is simply a special design that uses deep learning to anticipate and generate conversational language. Therefore, this presents a transformative opportunity for oncology nursing care planning. It could revolutionize decision-making, patient education, and care coordination, thereby elevating the standard of care provided.^3^ The incorporation of LLMs in nursing care planning is underpinned by the model's ability to analyze vast amounts of data rapidly and efficiently. GPT-4, for instance, can be trained to understand the latest evidence-based practices in oncology, which can facilitate timely and precise clinical decision-making.^4^ Consequently, nurses can provide personalized care based on the most up-to-date research and guidelines without manually sifting through the ever-growing medical and nursing literature.^5^ Additionally, LLMs can synthesize patient data and provide predictive insights. By integrating Electronic Health Records (EHRs), LLMs can assess the patient's history and current status and then predict potential complications or treatment responses, utilizing precise clinical decisions based on the retrieved information about the patient's condition.^6^ This enables the formulation of proactive care plans that address current needs and anticipate and mitigate future challenges. Moreover, patient education is an indispensable element of oncology nursing care planning. LLMs can generate tailored educational materials for patients and their families, adapted to their language proficiency and comprehension levels.^7^ Providing easy-to-understand information regarding their condition, treatment options, and self-care strategies empowers patients to participate in self-care management effectively, improving adherence and outcomes. Furthermore, LLMs can help streamline communication and coordination among multidisciplinary teams involved in the care of cancer patients.^8^ With their natural language processing capabilities, these models can facilitate the translation of complex medical jargon into layperson's terms or convert textual data into visual representations. This can enhance collaborative efforts by ensuring that all team members, including those from non-medical backgrounds, clearly understand the patient's condition and care plan.

To illustrate the application of LLMs in oncology nursing care planning, let us consider the hypothetical case of a 55-year-old female patient, Mrs. Jones, who was recently diagnosed with stage II breast cancer (Table 1). Upon diagnosis, Mrs. Jones' oncology nurse, Joyce, initiates a nursing care plan. Joyce uses an LLM integrated into the hospital's EHR system. The model quickly analyzes Mrs. Jones' medical history, family history of cancer, and current health status. It generates a summary and recommends evidence-based interventions based on the latest research. The model suggests that Mrs. Jones might benefit from specific nursing interventions to support her journey with cancer and highlights a recent study that supports this recommendation. Joyce reviews these insights and includes them in the care plan. Joyce then uses the language model to create personalized educational material for Mrs. Jones. The model generates information regarding her diagnosis, treatment options, potential side effects, and self-care strategies tailored to Mrs. Jones' educational background and comprehension level. Joyce reviews the material with Mrs. Jones, ensuring she understands her condition and the importance of adhering to her treatment plan. The LLM assists in keeping track of Mrs. Jones' development during her treatment journey. Joyce inputs Mrs. Jones' vital signs and laboratory results into the EHR, and the LLM analyzes these data to predict potential complications such as neutropenia. Joyce adjusts the nursing care plan accordingly, incorporating preventive measures and close monitoring of Mrs. Jones' white blood cell counts. In managing Mrs. Jones' care, Joyce coordinates with a multidisciplinary team, including oncologists, physical therapists, dietitians, and social workers. Joyce efficiently conveys critical information to the team members using the LLM's communication features. Visual representations of Mrs. Jones' progress and tailored summaries allow each team member to understand and contribute effectively to her care.

**Table 1:** An example of the incorporation of AI-assisted technology into oncology nursing care planning.

Steps	Example	Action by LLM/AI
Background	Mrs. Jones, a 55-year-old female, was recently diagnosed with stage II breast cancer. She has a family history of cancer and is anxious about her treatment and prognosis.	The LLM can also prompt relevant questions to gather a complete patient history such as asking about familial disease prevalence, lifestyle factors, mental health status, or medication adherence.
Step 1: Data collection	Oncology nurse Joyce collected comprehensive data including Mrs. Jones' medical history, family history, lifestyle, and mental health status. Vital signs, lab results, and diagnostic imaging reports were obtained and integrated into the hospital's EHRs.	The AI system processes and organizes the collected data, making it easily accessible for reference and analysis.
Step 2: Formulating nursing diagnoses	• Anxiety related to cancer diagnosis and treatment • Risk for Infection related to CTX-induced immunosuppression	The LLM suggests potential nursing diagnoses based on its analysis.
Step 3: Goals and expected outcomes	These include reducing Mrs. Jones' anxiety, preventing infections during treatment, and improving her understanding and management of her condition.	The model, based on evidence-based research, predicts expected outcomes and helps set SMART goals.
Step 4: Nursing interventions	• Individualized anxiety management • Infection prevention precautions and education	The model recommends several evidence-based nursing interventions
Step 5: Documentation and patient education material	Joyce documents the care plan and prepares patient education materials.	The AI helps Joyce document the care plan efficiently. Moreover, it generates patient education materials tailored to Mrs. Jones's condition and comprehension level.
Step 6: Evaluation criteria	An ongoing evaluation is conducted to monitor Mrs. Jones' response to the treatment and the efficacy of the nursing interventions.	The model uses continuous data input from Mrs. Jones' EHR to evaluate her response to the treatment and the efficacy of the nursing interventions. Joyce uses this information to adjust the care plan as needed, ensuring optimal patient outcomes and timely detection of potential complications.

CTX, Chemotherapy; HER, Electronic health record; LLM, Large language model; SMART, Specific, Measurable, Achievable, Relevant and Time-Bound; AI, artificial intelligence.

Skepticism towards artificial intelligence (AI) in healthcare stems from transparency issues, data privacy concerns, job displacement fears, and ethical quandaries.^9^ Lack of understanding about AI decision-making, concerns over the protection of sensitive patient data, fear of healthcare professionals' roles becoming redundant, and the ethical implications of AI usage contribute to mistrust. Overcoming this requires involving stakeholders in the decision-making process and developing clear protocols to review AI recommendations.^10^ This approach could address concerns, foster a sense of ownership, increase transparency, and build confidence in AI technology.

To summarize, harnessing LLMs like GPT-4 in oncology nursing care planning holds significant promise for improving the quality of care for cancer patients. This technology plays an important role in predicting silent trends in the given patient's data. Through enhanced decision-making, patient education, and care coordination, these models can lead to more personalized, proactive, and collaborative approaches, ultimately contributing to better patient outcomes.

## Data Availability

Data availability is not applicable to this article as no new data were created or analyzed in this study.

## CRediT author statement

AJN & SBH: Conceptualization, writing – original draft, editing, review – final draft. All authors read and approved the final manuscript.
